# Mandatory First-Aid Training in the Workplace: An Epidemiological Assessment of the Use of Acetylsalicylic Acid Therapy

**DOI:** 10.3390/epidemiologia6030049

**Published:** 2025-09-01

**Authors:** Elena Maria Ticozzi, Nazzareno Fagoni, Erika Kacerik, Annalisa Bodina, Gabriele Perotti, Massimo Lombardo, Fabrizio Ernesto Pregliasco, Giuseppe Stirparo

**Affiliations:** 1 Department of Biomedical Sciences for Health, Università Degli Studi di Milano, 20122 Milano, Italy; 2 Agenzia Regionale Emergenza Urgenza (AREU), 20124 Milano, Italy; 3 Department of Molecular and Translational Medicine, University of Brescia, 25123 Brescia, Italy; 4 School of Medicine, Università Vita e Salute San Raffaele, 20132 Milan, Italy

**Keywords:** occupational health, STEMI, myocardial infarction, emergency medical system

## Abstract

**Background**: In Italy, workplace safety regulations require the training of first-aid officers to manage medical emergencies, including acute coronary syndromes. Although clinical guidelines recommend the early use of acetylsalicylic acid in myocardial infarction, little is known about the implementation of this recommendation in practice. This study aims to assess the use of acetylsalicylic acid for ST-elevation myocardial infarction (STEMI) in workplace and non-workplace settings, with a focus on informing the evaluation and improvement of first-aid training programs and emergency response protocols. **Methods**: We conducted a retrospective, observational cohort study using 2019 data from the Regional Agency for Emergency Urgency. Cases were identified and stratified by event location (workplace vs non-workplace), to analyze patterns of acetylsalicylic acid administration. A logic model has been developed to program a stepwise plan of action for policies development. **Results**: A total of 2174 STEMI cases were identified, of which 380 (17.5%) occurred in the workplace. Workplace cases were younger and more likely to be male. Acetylsalicylic acid was administered in only 31 cases overall, with no statistically significant difference between settings. This assessment advocates for the implementation of targeted actions, which may include updates to current legislation and policies. **Conclusions**: These findings highlight an urgent need to systematically evaluate existing workplace first-aid training and emergency protocols. Integrating modules on acetylsalicylic acid administration into training curricula, along with performance monitoring mechanisms, may significantly enhance early STEMI management and patient outcomes. Updating safety programs to align with evidence-based practices should follow a structured approach.

## 1. Introduction

First-aid training is an essential element of a safe workplace, since it equips workers with the skills necessary to respond quickly to medical emergencies, potentially saving lives and minimizing the severity of sudden injuries or medical emergencies. Immediate assistance can be critical in reducing recovery time and preventing complications of several pathologies. This kind of training could also promote a safer work environment, as employees become more aware of potential health hazards, making them better prepared to assist co-workers or customers in need of help. Given that most people spend a significant portion of their day at work, first-aid skills and emergency preparedness are critical, as emergencies can occur at any time.

The European Agency for Safety and health at Work guide describes first aid as the initial emergency care for injuries or sudden illness before the arrival of emergency medical services [1]. According to their statement, workplace first-aid providers should be trained to perform basic assessments and interventions, taking into account the limited equipment at their disposal. According to the European recommendations, employers should designate a specific person to manage the occurrence of workplace emergencies and to select and maintain first-aid supplies, ensuring that they are appropriate for potential injuries in the workplace and are stored in an easily accessible area [1].

In Italy, first aid in the workplace is regulated by the Legislative Decree 81/2008, which establishes that the employer must designate a worker in the company as a first-aid operator, whose role is to provide first aid and recognise situations that require the intervention of the emergency system [2]. To this end, the designated employee must receive appropriate training in the recognition and treatment of emergencies. According to the most recent data, the number of first aiders in Italian companies has increased over the years, exceeding the legal requirements [3]. This phenomenon is possibly linked to an increased perception of the importance of first-aid training by both employers and employees. The Legislative Decree 81/2008 provides for a series of mandatory training modules covering the management of emergencies such as out-of-hospital cardiac arrest, anaphylactic shock, the recognition of medical emergencies such as myocardial infarction and stroke, and the management of first aid in cases of trauma. The training modules alternate practical and theoretical activities to give a comprehensive overview of the required actions. In addition to the training required by the Legislative Decree 81/2008, some workplaces require a different specific training regulated by Legislative Decree 158/2012, which includes specific requirements for sports hall operators, with a particular focus on the management of out of hospital cardiac arrest and the use of defibrillators [4,5]. Numerous studies have shown that practical and theoretical training in emergency management reduces patient mortality and increases the possibility of correctly managing a medical emergency, improving clinical outcomes [6,7]. Due to the extreme importance of early recognition and treatment of myocardial infarction, the Italian Legislative Decree 116/2021 expanded the training requirements and the list of workplaces in which the purchase of defibrillators is mandatory [8].

One of the emergencies that must be handled by the first responders is the management of ST-elevated myocardial infarction, as stated by ministerial indications. Their task is to recognize the specific signs and symptoms associated with ST-elevated myocardial infarction and to promptly alert the emergency system [2]. STEMI is a time-dependent pathology that benefits from early intervention through the delivery of reperfusion therapy, therefore early recognition and management are vital to reduce the time from symptoms onset to treatment. Patients with ST-elevated myocardial infarction typically present with symptoms such as chest pain, shortness of breath, nausea, and sweating, allowing for early recognition; the emergency system operators can confirm the diagnosis by performing an electrocardiogram (ECG) in patients with suggestive signs and symptoms [9]. Early diagnosis at a prehospital level is crucial because it allows the emergency medical system to alert the hospital of destination and to transfer the patient to a centre with adequate resources with the goal of performing the definitive treatment within 120 min from the first medical contact. Furthermore, an early diagnosis ensures a better prognosis for the patient, avoiding the possible evolution into cardiac arrest, decreasing mortality and morbidity [10,11]. In fact, the management of this medical emergency has improved over the years with the implementation of territorial management protocols and with the adoption of standardized hospital procedures and therapies, with the aim of achieving reperfusion as early as possible in each patient [10].

Early out of hospital management of ST-elevated myocardial infarction includes the administration of antiplatelet agents as a rapid first-line treatment, in order to reduce mortality and post-treatment complications [12,13,14,15,16]. Some antiplatelet agents, such as acetylsalicylic acid, are also commonly used in the primary prevention of cardiovascular diseases [17], making them one of the most widely available drugs. According to the European Resuscitation Council 2023 guidelines and to the American Heart Association protocol, treatment with acetylsalicylic acid in ST-elevated myocardial infarction should be started as soon as possible with an oral dose of 160–325 mg of non-enteric coated chewable acetylsalicylic acid. This therapy should be administered to all the patients except those who are allergic to it or those who have experienced gastric haemorrhage in the previous three months [9,18,19,20].

In Italy, a large proportion of the working population is trained in the management of medical emergencies due to the presence of mandatory courses required by law [3,21]. Widespread first-aid training increases the likelihood of early recognition and correct bystander management of medical emergencies in the workplace compared to other settings, but there is still a lack of knowledge regarding the use of therapy with acetylsalicylic acid [22]. Regarding the management of ST-elevated myocardial infarction, the set of skills taught to first-aid responders in the workplace comprehend the early recognition of symptoms, but, unlike other pathologies such as anaphylactic shock, this training does not include first treatment notions; therefore first responders are not trained to administer initial supportive drug therapy with acetylsalicylic acid. According to international guidelines, early therapy with acetylsalicylic acid can be safely administered under the supervision of medical personnel on the phone while waiting for emergency services to arrive on the location of the event. At a European level there are inconsistent regulations regarding the presence of acetylsalicylic acid in first-aid kits. While generally medications are not included in first-aid kits, the United Kingdom explicitly states that acetylsalicylic acid tablets could be an exception, as they are mentioned in current chest pain guidelines [23]. No country currently mandates the inclusion of medications in standard workplace first-aid kits. The topic remains under debate, with differing national guidelines and practices regarding the optional presence of over-the-counter drugs in occupational settings [1,23].

A possible explanation for the exclusion of acetylsalicylic acid from workplace emergency kits is that the administration of drugs, including ASA, is generally considered a medical act and may raise liability concerns in the absence of medical personnel. Furthermore, the risk of adverse reactions (including allergic reactions, bleeding complications, or interactions with other medications) may contribute to this approach.

The aim of this study is to evaluate the current implementation and potential for promoting the early use of acetylsalicylic acid in the management of ST-elevation myocardial infarction, particularly in workplace settings. To this end, we analysed data from the prehospital emergency medical system in the Lombardy region during 2019, focusing on demographic and clinical characteristics of ST-elevated myocardial infarction cases and comparing administration of acetyl salicylic acid between workplace and non-workplace scenarios. This evaluation was intended to provide support for the development of targeted policy and training interventions aimed at increasing adherence to clinical guidelines.

## 2. Materials and Methods

This is a retrospective observational cohort study. The following study adheres to the STROBE guidelines for cohort studies [24].

### 2.1. Emergency Medical System in Lombardy

Lombardy is Italy’s largest and most populous region, with an area of 23,863 km^2^ and a population of 9.96 million inhabitants. In Lombardy, the prehospital emergency system is managed by the Regional Emergency Urgency Agency (AREU), which coordinates all the emergency requests using wheeled vehicles or helicopters [25,26]. In the event of a medical emergency, citizens can dial the European emergency number 112 and speak to a trained operator who assesses the severity of the emergency by means of branched tree questions, assigning a severity code (white, green, yellow, or red) to optimize the use of resources and vehicles. The logistics of the rescue missions are coordinated by regional dispatch centres, which identify the most appropriate vehicle to send to the scene and the most appropriate hospital to transport the patient according to the type and severity of the emergency. There are different types of wheeled vehicles with different team composition: the dispatch centre operator can choose to send a basic vehicle, in which there are only trained volunteers, or an advanced vehicle with a nurse or with a nurse and a medical doctor. All rescue missions are recorded in real time on the SAS-AREU portal, which contains logistical and medical details of each patient rescued by the emergency medical system [27,28].

### 2.2. Data Registry

Data were provided by the register of the Lombardy AREU headquarters and extracted from the SAS-AREU database, which contains all data on emergency calls to the prehospital emergency system, with the clinical and logistical details of the rescue missions. Data were collected and analysed for the time period from 1 January 2019 to 31 December 2019. Data regarding all related scenarios were selected and extracted accordingly.

For this purpose, we analysed data regarding the number of ST-elevated myocardial infarction diagnoses, the average time of arrival of the first emergency vehicle at the scene, the average transport time from the scene to the hospitals, and the location of the rescue event. “Workplace ST-elevated myocardial infarction” was defined as an event occurring at selected locations characterized by the presence of workers, including railways, sports facilities, schools, and public offices, while all other rescue locations were classified as “non-workplace ST-elevated myocardial infarction”.

### 2.3. Statistical Analysis

Categorical variables are presented as numbers. Continuous variables are presented as mean and standard deviation (SD). Categorical variables were analysed using the χ^2^ test. Continuous variables were tested for normality using the Kolmogorov–Smirnov test, and the Z test was performed to analyse the means. Differences were considered significant when *p* < 0.05; otherwise, they were considered non-significant. The statistical software Prism 8.0.1 (GraphPad Software LLC, San Diego, CA, USA) was used for this purpose.

### 2.4. Logic Model Development

We developed a logic model following the Centers for Disease Control Division for Heart Disease and Stroke Prevention recommendations for public health program planning with the aim to guide the design, implementation, and monitoring of possible future actions [29]. The adopted logic model begins with the identification of current inputs, proposed actions and outputs, and expected outcomes. The final aim of this approach is the effective integration of evidence from clinical guidelines into current policies.

## 3. Results

In line with the scope of this research, we adopted a logic model as suggested by the CDC’s Division for Heart Disease and Stroke Prevention [29]. As a first step, we conducted an analysis of the use of acetylsalicylic acid therapy to assess how current clinical practice deviates from the guidelines. In a second step, we developed a workflow proposal to guide the improvement of current guidelines to reduce the potential disability and mortality associated with the condition.

### 3.1. Assessment of Current Clinical Practice

In 2019, a total of 821,689 missions were completed, and 84,073 ECG were performed by emergency teams, of which 2174 were identified as ST-elevated myocardial infarction by the prehospital emergency system. The mean age of patients was 66.1 years (SD: 13.4), and the number of male patients was 1547 (71.2%). Table 1 shows the location of the patient’s rescue; 380 (17.5%) cases occurred in the workplace and were categorized as “workplace ST-elevated myocardial infarction”, while the remaining 1794 (82.5%) cases occurred in a different setting and were accordingly categorized as “non-workplace ST-elevated myocardial infarction”, as showed in Table 1.

Of the total number of patients diagnosed with ST-elevated myocardial infarction, 765 (35.2%) were assigned a red code, 1357 (62.4%) were assigned a yellow code, and the remaining 52 (2.4%) had a green code. A total of 25.0% (543) of the calls were handled by a vehicle staffed with a trained nurse, while 57.9% (1258) of the calls were handled by a vehicle staffed with a physician. A total of 8% (173) of cases were supported by both type of medical vehicles, while 9.2% of calls (200) were handled by a basic support vehicle staffed by volunteers.

Table 2 shows the demographic characteristics of the population analysed. The proportion of males experiencing ST-elevated myocardial infarction in the workplace is significantly higher than in other locations (77.1% vs. 68.7%; *p* value < 0.01). Furthermore, patients experiencing ST-elevated myocardial infarction at the workplace are younger than those experiencing it at non-workplace locations, with a mean age of 63.0 years compared to a mean age of 67.8 years (*p* < 0.001).

Considering the average time of arrival of the first emergency vehicle at the rescue scene, it is slightly shorter when the event occurs at the workplace than at other locations (11.4 vs. 13.0 min; *p* < 0.01). Furthermore, the arrival time at the hospital is also onfimr shorter for cases happening at the workplace compared to those at non-workplaces (54.3 vs. 58.5 min; *p* < 0.01).

Only 31 subjects (1.4% of the total analysed population) used first-line therapy with self-administration of acetylsalicylic acid. The mean age of these patients was 64 years (SD: 10), of whom 20 were males. Regarding the location of the events of these 31 patients, seven occurred at the workplace and twenty-four happened in other contexts. Patients who assumed ASA in the workplace did so under the supervision of first-aid officers. The probability to receive a first therapy with acetylsalicylic acid at workplace was not different than to receive it in other settings (OR 1.4; IC 95% 0.6–3.2; *p* = 0.45).

### 3.2. Logic Model Framework for Guiding Policy and Program Improvements

We present the developed logic model framework in Table 3, with the aim to propose a stepwise action plan to integrate the current policies.

The inputs, which include comprehensive regional emergency medical services data from our agency, Italian and European legislation, and clinical guidelines, provide a solid foundation. However, a key limitation is the limited integration between these areas, with existing legislation not explicitly addressing pharmacological first aid in non-clinical settings.

The proposed activities are deemed feasible. Indeed, the first step of our study already addressed the needs assessment and policy evaluation part of the proposed outputs. However, the modification of current workplace first-aid kits would require an integration of the normative, which could require time and effort as these interventions may face resistance due to long timing or logistical concerns about including medicines in standard workplace kits. Nonetheless, the proposed outputs are aimed at delivering concrete changes in workplace preparedness and first aid, to align interventions with international best practice guidelines.

Regarding short and intermediate outcomes, the model anticipates an increase in awareness, earlier therapeutical administration, and better adherence to clinical protocols. These outcomes are highly dependent on a consistent quality of training for first-aid workers, which would require an updated module in the current curricula mandated by law. The expected long-term outcomes include a reduction in morbidity and mortality of ST-elevation myocardial infarction.

## 4. Discussion

This study shows a significant underuse of therapy with acetylsalicylic acid in cases of ST-elevated myocardial infarction, both in the workplace and in other settings. Indeed, our study reported that acetylsalicylic acid was used in only 1.4% of cases, with no significant difference between workplace and non-workplace locations.

However, it must be emphasized that in Lombardy most cases were handled by an advanced medical vehicle: 25.0% of the cases were managed by a vehicle staffed with a nurse, while in 60% of the cases a vehicle with a physician was present at the scene. According to the out-of-hospital ST-elevated myocardial infarction management protocols, the prehospital emergency teams in advanced vehicles administer intravenous/oral antiplatelet therapy, compensating the missed administration of oral acetylsalicylic acid. The remaining 9.2% of cases were handled by vehicles staffed exclusively by volunteers, who perform an ECG based on the patient’s symptoms and the indications of the medical doctor present in the dispatch centre. The ECG is evaluated in real time by the physician of the dispatch centre. In these cases, however, the volunteers cannot administer drugs, so the patient could benefit from the self-administration of acetylsalicylic acid, under the instructions of the dispatch centre physician.

The developed logical model proposes a series of actions to increase the use of acetyl salicylic acid. In order to facilitate access to this treatment, it could be beneficial to modify the first-aid course provided for by Legislative Decree 81/2008, adding a focus on the use of acetylsalicylic acid to the module teaching the recognition of the symptoms of ST-elevated myocardial infarction. At the same time, it would be necessary to update the mandatory composition of first-aid kits by adding chewable acetylsalicylic acid tablets to the list of their components. In Italy drugs cannot be administered by non-healthcare personnel, even those belonging to the emergency system; however, they could be instructed to allow the patient to take the drug autonomously after a brief discussion with a dispatch centre physician. This training could be particularly relevant in cases that are managed by a vehicle staffed by volunteers, without the support of a medical team.

Considering the expected outcomes, Legislative Decree 81/2008 is also considered a source of information by the general public; therefore, it would be reasonable to expect an increase in access to this treatment even in all settings.

Early administration of acetylsalicylic acid is an important step in the early management of STEMI, especially when considering the reported long average time to hospital arrival of 54.3 min from the workplace and of 58.2 min in other settings. Indeed, in cases of non-medical transport, early administration of acetylsalicylic acid prior to arrival at the hospital could improve patient outcomes. Our results highlighted that the time employed by the first emergency vehicle to arrive at the scene was shorter for workplace STEMI than for non-workplace STEMI (11.4 vs. 13.0 min for work and non-workplaces, respectively). This difference is probably a consequence of the fact that workplaces tend to be located on more accessible transportation routes than other locations, making them easily accessible by emergency vehicles.

We observed significant demographic differences between patients rescued in the workplace and those rescued in other contexts, with a higher percentage of males and a lower mean age in workplace cases. Two considerations could explain these findings: in Italy, a higher percentage of men are employed compared to women, and a large proportion of people retire after the age of 67 years old; therefore, older patients tend to be no longer employed. While older men are considered more prone to acute cardiovascular events, recent studies demonstrated that STEMI has a high impact on the female population as well, although it may present with different signs and symptoms, making it more difficult to recognize in the prehospital setting [30,31,32,33]. These events have a strong social impact [34]. Indeed, it would be of interest to further study and characterize the population suffering of STEMI in the workplace, to address possible gender-biases in the current content of training.

A limitation of our study is that we cannot assess whether the first-aid treatment was primarily given to workers or non-workers; in fact, we analysed the events based on location, but we cannot assess whether the rescue was requested for a client or guest inside the structure or for a worker. At the same time, we do not know whether the activation of the 112 system at the scene of the event was performed by the trained first-aid operator or by other bystanders present at the scene. Another possible limitation is that the number of patients identified as STEMI by the prehospital emergency system is limited to those cases in which the presence of typical signs and symptoms mandated the performance of a prehospital ECG, missing cases with atypical presentations or with a first negative ECG are identified as STEMI upon their arrival at the hospital.

On the other hand, the analysed database includes all the rescue operations carried out in the Lombardy region over the course of one year, giving an accurate vision of the management of STEMI recognized as such by the prehospital emergency system, including the correct activation and use of guidelines by bystanders at the first scene of the event.

According to our findings, we suggest that the results of this study could potentially pave the way for future actions, starting with an update in mandatory workplace training and first-aid kit composition. Although we analysed data from 2019, it would be interesting to evaluate the evolution of STEMI in the workplace after the COVID-19 pandemic, as its unprecedented impact prompted the adoption of more flexible work-from-home policies by several companies, changing the physical location of work with different consequences. Indeed, policy makers should consider the constant evolution of work, following the principle that it should be performed in a safe environment.

## 5. Conclusions

Despite the presence of recommendations in different international guidelines, the use of a first therapy with acetylsalicylic acid in patients with symptoms of STEMI is underused, both in the workplace and in other settings. To increase its use, it could be beneficial to implement specific prevention projects and to extend the training provided for by the Legislative Decree 81/2008, to increase the awareness of the general population and of workplace first-aid operators about this topic.

While the number of patients with STEMI rescued at the workplace is a minority compared to the total number, in these rescue missions we registered a shorter time for the first vehicle to arrive at the scene and a shorter time to arrive at the hospital, possibly since workplaces are in more accessible locations. Those rescued at the workplace have a lower mean age and a higher percentage of males than in other locations, suggesting that further research is needed to identify specific public health interventions that could be beneficial to improve STEMI management in this specific population and to address possible gender-biases in the recognition of STEMI at the workplace.

The results of this study show a significant underuse of acetylsalicylic acid therapy in STEMI cases, both in the workplace and in non-workplace settings. This highlights a wider lack of awareness in the general population, which is compounded by gaps in legislation regarding first-aid policies and the contents of first-aid kits. These findings have important practical implications for public health professionals and policy makers. Where appropriate, public awareness should be increased through targeted educational campaigns, and first-aid policies should be updated to bring them more in line with current clinical guidelines.

## Figures and Tables

**Table 1:** Location of recorded ST-elevated myocardial infarction (STEMI) events in 2019, divided into workplace and non-workplace events.

Workplace Locations	Number of STEMI	Percentage	Total
Other healthcare facilities	62	2.9%	380 (17.5%)
Railways	4	0.2%	
Work facility	65	3.0%	
Sport facility	25	1.2%	
Hospital	11	0.5%	
School	5	0.2%	
Residential facility	51	2.3%	
Offices	157	7.2%	
**Non-workplace**			
Home	1623	74.7%	1794 (82.5%)
Mountains	7	0.3%	
Road	153	7.0%	
Undefined	11	0.5%	

**Table 2:** Demographic characteristics of patients and mean times of rescue missions, in cases of ST-elevated myocardial infarction (STEMI) happening at workplace or in other locations. § Statistical analysis with chi-square test; * Statistical analysis with Z-test for two means.

	Workplace STEMI	Non-Workplace STEMI	*p* Value
Males (%)	220 (85%)	1327 (69%)	<0.01 ^§^
Females (%)	87 (22.9%)	666 (37.1%)	<0.01 ^§^
Age (SD)	58 (11)	67 (13)	<0.01 *
Mean time first vehicle min (SD)	11 (5)	13 (6)	<0.01 *
Mean arrival time in hospital min (SD)	54 (19)	58 (20)	<0.01 *

**Table 3:** Logic model proposal to promote use of acetylsalicylic acid in workplace response. Abbreviations: ESC (European Society of Cardiology); AHA (American Heart Association); EMS (Emergency Medical Services); STEMI (ST-elevated myocardial infarction).

Component	Details
Inputs	- Prehospital myocardial infarction data from Lombardy region - Current legislation - Clinical guidelines (ESC/AHA) - First-aid course curricula
Activities	- Needs assessment (acetylsalicylic acid use gap) - Policy and legislation analysis - Development of revised training and kit standards
Outputs	- Updated training modules with acetylsalicylic acid content - Revised first-aid kits including chewable acetylsalicylic acid - Supervised self-administration via EMS call
Short-term Outcomes	- Increased awareness among workplace first responders - Higher rates of acetylsalicylic acid use in early STEMI management in all settings
Intermediate Outcomes	- Reduced delay in acetylsalicylic acid administration - Improved alignment with international guidelines
Long-term Outcomes	- Reduced STEMI-related morbidity and mortality

## Data Availability

Data are available upon reasonable request.
